# Supplementary material for: Dynamics of the Emerging Genogroup of Infectious Bursal Disease Virus Infection in Broiler Farms in South Korea: A Nationwide Study
Source: Viruses. 2022 Jul 22;14(8):1604. doi: 10.3390/v14081604 (PMC9330851; doi:10.3390/v14081604)
Supplement: Supplementary file 1 [file viruses-14-01604-s001.zip › viruses-1818977-supplementary.pdf]

Table S1. Sampling scheme and sample size calculation of broiler chicken farms for the prevalence estimation of infectious bursal disease virus infection in South Korea.

Poultry abattoir (n=43)	Annual number of chickens slaughtered in South Korea (year of 2019)	Proportion of chickens slaughtered over total number (%)	Designed number of samples collected (unit: farm)	Actual number of samples collected (unit: farm)
PA1	21,546,591	2.033	4	4
PA2	7,260,884	0.685	1	0
PA3	41,050,326	3.873	8	8
PA4	3,044,571	0.287	1	2
PA5	20,873,127	1.969	4	1
PA6	18,538,596	1.749	3	3
PA7	431	0.000	0	0
PA8	12,238,989	1.155	2	2
PA9	11,828,326	1.116	2	2
PA10	29,499,580	2.783	6	6
PA11	165,869	0.016	0	0
PA12	12,929,581	1.220	2	2
PA13	4,601,896	0.434	1	1
PA14	26,533,081	2.503	5	5
PA15	36,425,195	3.436	7	6
PA16	61,365,268	5.789	12	16
PA17	11,908,655	1.123	2	2
PA18	18,373,046	1.733	3	0
PA19	28,167,972	2.657	5	1
PA20	18,400,709	1.736	3	3
PA21	10,298,101	0.972	2	1
PA22	36,106,777	3.406	7	7
PA23	15,322,968	1.446	3	0
PA24	12,909,898	1.218	2	4
PA25	87,656,887	8.270	17	16
PA26	136,083	0.013	0	0
PA27	85,297,580	8.047	16	16
PA28	5,575,628	0.526	1	0
PA29	121,532,115	11.465	23	24
PA30	76,079,527	7.177	14	11
PA31	31,565,436	2.978	6	0
PA32	16,677,580	1.573	3	0
PA33	1,951,691	0.184	0	0
PA34	20,896,004	1.971	4	4
PA35	19,603,687	1.849	4	0
PA36	20,153,242	1.901	4	0
PA37	1,622,197	0.153	0	0
PA38	90,804,344	8.566	17	17
PA39	7,790,182	0.735	1	1
PA40	3,103,838	0.293	1	0
PA41	1,659,525	0.157	0	0
PA42	3,924,415	0.370	1	1
PA43	4,574,092	0.432	1	1
Total	1,059,994,490	100.000	200	167

* Designed number of samples collected in each abattoir (unit: farm) was calculated by multiplying the proportion of chickens slaughtered over the total number of chickens slaughtered across the country with designed total sample size (200).
